# Adolescents’ Food Purchasing Patterns in The School Food Environment: Examining the Role of Perceived Relationship Support and Maternal Monitoring

**DOI:** 10.3390/nu12030733

**Published:** 2020-03-11

**Authors:** Roel C.J. Hermans, Koen Smit, Nina van den Broek, Irma J. Evenhuis, Lydian Veldhuis

**Affiliations:** 1 The Netherlands Nutrition Centre, 2594 AC The Hague, The Netherlands; evenhuis@voedingscentrum.nl (I.J.E.); veldhuis@voedingscentrum.nl (L.V.); 2 Department of Health Promotion, NUTRIM School of Nutrition and Translational Research in Metabolism, Maastricht University, 6299 AH Maastricht, The Netherlands; 3 Behavioural Science Institute, Radboud University, 6500 HE Nijmegen, The Netherlands; k.smit@bsi.ru.nl (K.S.); n.vandenbroek@bsi.ru.nl (N.v.d.B.); 4 Trimbos Institute, Netherlands Institute of Mental Health and Addiction, 3500 AS Utrecht, The Netherlands

**Keywords:** school food environment, dietary behavior, food purchasing, adolescents, maternal monitoring, perceived relationship support

## Abstract

The school food environment plays a role in adolescents’ dietary behaviors. In this study, adolescents’ food purchasing patterns in and around school and its potential relationship with perceived maternal relationship support and maternal monitoring were examined. Data were collected in The Netherlands in 2017. A total of 726 adolescents (45.8% boys; *M*_age_ = 13.78 *±* 0.49) and 713 mothers (*M*_age_ = 45.05 ± 4.45) participated. Adolescents’ frequency of bringing and purchasing foods was assessed via a Food Frequency Questionnaire (FFQ). Relationship support and monitoring were measured via self-report questionnaires. Structural Equation Modelling (SEM) was conducted to examine associations between adolescents’ food purchasing patterns, relationship support, and monitoring. Results indicated that adolescents brought food and drinks mostly from home, and infrequently purchased these products in and around school. Yet, differences exist between subgroups of adolescents. Relationship support was positively associated with bringing fruit, vegetables and salad and negatively associated with purchasing sweet snacks. No associations were found for monitoring. These findings indicate that family-home determinants of healthy and unhealthy eating are important factors to consider when examining the impact of the school food environment on adolescents’ food purchasing patterns. This has implications for policy makers who aim to develop and implement measures to improve adolescents’ eating in and around school.

## 1. Introduction

The prevalence of overweight (including obesity) in children and adolescents in the WHO European region is alarming and considered to be one of the most serious public health challenges of the 21st century [1]. Globally, one in five children and adolescents aged 5–19 years have overweight, with levels increasing rapidly in many countries and regions in recent years [1]. The Netherlands is no exception in this case, with almost 12% of the children aged 4 to 17 years considered to have overweight in 2018 [2]. Healthy eating throughout the life-course helps prevent malnutrition in all its forms, as well as the development of a range of non-communicable diseases such as diabetes and heart disease. Furthermore, it is crucial for ensuring optimal physical and cognitive development during childhood and adolescence [3]. Yet, many children and adolescents do not meet dietary intake guidelines: they consume high levels of added sugar and/or fat, and have low intakes of fruits, vegetables, and whole grains [4,5,6]. Excessive consumption of energy-dense, nutrient-poor foods and drinks are a key cause of weight gain and contribute substantially to the development of overweight and obesity among youth [7]. Understanding the drivers of these unhealthy eating patterns is therefore essential to inform targeted approaches for overweight prevention in youth.

The current food environment, in particular, has been proposed to contribute importantly to the sharp increase in obesity rates worldwide [8,9,10]. As a consequence, there is also increasing attention to the role of the school food environment on young people’s dietary behaviors (e.g., [11,12,13]). Adolescents are offered a variety of eating options and opportunities within their schools, varying from national lunch programs to food retailing in canteens and vending machines. In recent years, efforts have been made to create healthier school food environments [14,15,16,17]. These preventive school-based programs often focus on improving the nutritional quality of existing lunch meal programs or the food and beverage assortment in canteens and vending machines, thereby increasing the availability of healthy foods and limiting the supply of unhealthy foods. Furthermore, nudging and social marketing strategies are employed to steer adolescents towards better food choices by modifying the direct school food environment (e.g., by providing ready access to potable water or displaying fruits and salads in attractive bowls or stands). There is also a growing body of literature that recognizes the role of the local retail food environment around schools. There are studies that demonstrate, for instance, that a high fast-food outlet density in school neighborhoods is associated with increased fast-food purchasing by adolescents [18] or decreased odds of daily fruit and vegetable intake [19]. Furthermore, given that studies show that unhealthy food options (e.g., fried snacks and sugar-sweetened beverages) are more often for sale, in-store promoted or advertised in comparison with healthy options (e.g., fruit or bottled water [20]), these outlets in school neighborhoods are a key competitor of (healthy) school canteens, particularly since adolescents feel that they can get lower prices, more variety and more value for money in these outlets [21,22].

As research exploring the associations between school environments and adolescents’ food purchasing behavior in European countries is sparse, the first aim of this study was to acquire more insight into the frequency at which Dutch adolescents purchase food and drinks in their school food environment. Previous work conducted in The Netherlands demonstrated that adolescents mostly bring food and drinks from home as lunch, whereas the school canteen is primarily visited to buy something extra [21,22]. Adolescents also buy their food and drinks at nearby food retailers, whenever the school permits them to leave school grounds. Demographic factors, such as adolescents’ sex, age and educational level, play a role in their food purchasing behaviors. Older adolescents, for instance, may be more likely to make food purchases in and around school. Considering the potential role of these factors in adolescents’ food purchasing behaviors, we also examined how these factors (i.e., adolescents’ sex, age, educational level, and Body Mass Index (BMI)) are associated with adolescents’ frequency of food purchasing in the school food environment. Specifically, we examined food purchasing behavior in four different food/beverage categories; fresh fruit, vegetables and salad (FVS); sugar-sweetened beverages (SSB), sweet snacks (SWS) and savory snacks (SAS), as these are associated with health promotion and disease prevention [3]. In particular, positive associations have been found between consumption of ultra-processed food and body fat during childhood and adolescence [23].

The school food environment is not the only factor that has an influence on adolescents’ food purchasing patterns in and around school. Parents also play an important role in adolescents’ food attitudes and behaviors. This can occur through various processes such as their own dietary intake patterns and their food-related parenting practices [24]. A recent systemic review, for instance, demonstrated that the availability of healthy foods and non-availability of unhealthy foods are associated with decreased unhealthy eating in adolescents [25]. Likewise, the effects of parental modeling on adolescents’ dietary behavior have been found to be consistent and significant across studies; the frequency at which parents eat healthily and demonstrate the benefits and pleasure of eating healthily are associated with adolescents’ healthy eating patterns [26]. Other food parenting practices, such as setting restrictions regarding food consumption or food monitoring, have not been found to be consistently related to adolescents’ dietary behavior [26]. Inconsistent findings in this domain are often explained by the influence of general parenting [27], which is defined as the emotional climate in which specific parenting practices are expressed [28]. For example, it has been demonstrated that the use of parenting practices such as encouragement and covert control led to an increase in healthy intake and decrease in unhealthy food intake only in those children who were reared in a positive parenting context (characterized by parental warmth and guidance) [29]. As such, authoritative parenting is often found to be associated with better weight-related outcomes compared to other parenting styles such as permissive or coercive forms of parenting [27,30]. As research on the role of parental factors in explaining adolescents’ food purchasing in the school food environment is scarse, a second aim of the present work was to acquire more insight into the frequency at which adolescents bring food and drinks from home. Again, we also examined how demographic factors are associated with this behavior. Finally, the present research examined for the first time whether two specific parenting factors (i.e., perceived relationship support and monitoring) were associated with adolescents’ frequency of bringing and purchasing food and drinks in the school food environment. Data on the extent to which adolescents received relationship support from their mother and mothers’ knowledge about their child’s daily activities (i.e., maternal monitoring) were collected in a previous wave of a longitudinal lifestyle cohort study [31,32]. This final research aim, therefore, is explorative in its ambition, without having derived causal hypotheses.

## 2. Materials and Methods

### 2.1. Procedure and Materials

Data for the current study were drawn from a multi-informant, seven-wave longitudinal lifestyle cohort study (2015–2018). We aimed to recruit a nationwide representative sample of 10-to-13-year-olds and their mothers. Therefore, random sampling methods were used to recruit participants from five randomly selected regions and provinces in the Netherlands [31,32]. The regions were based on the four cardinal points (i.e., North, East, South, West). The center of The Netherlands was added as an extra region, resulting in a total of five regions. After distributing the twelve Dutch provinces across these regions, we randomly selected one province in each of the five regions using the website www.random.org. We then retrieved a list of all primary schools in these provinces via the website of Dutch Ministry of Education (*n* = 913). Management boards of these schools were then contacted by telephone. Of the 913 schools, 123 school boards agreed to participate (13.5%). After providing consent, schools were asked to distribute invitation letters to children in the 6th grade of primary education. Children and mothers from 104 of the 123 participating schools opted into the study. To register for the study, mothers (at baseline, *n* = 755) and their children (*n* = 755) had to provide informed consent through the research project’s website. All participants were informed that their participation was voluntary, and that they could withdraw from the study at any time. The current study was conducted in accordance with the Declaration of Helsinki, and the study procedures were approved by the ethics committee of the Faculty of Social Sciences of Radboud University, Nijmegen, The Netherlands (ECSW2014-2411-272). 

At baseline, paper and pencil questionnaires were administered to students in the classroom. In the same week, mothers were requested to complete the online questionnaire by e-mail. In the following three years, online questionnaires were sent to the adolescents every six months. Their mothers received their questionnaire via e-mail every twelve months. Yearly monetary incentives (€10) were provided to both adolescents and their mothers. If the mother was not available for any reason, the father could complete the questionnaires. However, these cases were excluded in the present study. The questionnaire used in this study is available in Dutch from the corresponding author upon request.

### 2.2. Participants

The current study used fifth wave data (2017), including 726 adolescents (46% boys, *M*_age_ = 13.78) and 713 mothers. Self-reported information about length (in centimeters) and weight (in kilograms) was provided by 72.8% of the adolescents, and by 91.4% of the mothers. Adolescents lived together with two parents (79%) or within a divorced household (21%). Adolescents were almost equally distributed over the three educational levels (i.e., low-medium-high) in The Netherlands (see Table 1).

### 2.3. Measures

All of the measures relevant to the present research are outlined in detail below. Measures related to adolescents’ food purchasing behavior were specifically added to the fifth wave of the longitudinal lifestyle cohort study. All the other measures were already part of the existing cohort study and also measured at the fifth wave, unless otherwise specified.

*Demographic information adolescent*. Adolescents’ sex and birth date were assessed at baseline, their weight and height and their educational level at wave five. Adolescents’ age was obtained from their reported date of birth and the date of measurement. Adolescents reported on their height in centimeters and their weight in kilograms. To calculate adolescents’ zBMI-for-age, first adolescents’ BMI was computed by dividing their weight in kilograms by their squared height in meters. Subsequently, adolescents’ zBMI-for-age was computed by considering the age- and gender-specific growth curves for BMI, based on a Dutch representative sample of 0-to-21-year-olds [33]. Adolescents’ educational level was measured with one item (‘what is your level of education?’), and response categories ranged from the lowest to the highest educational level in The Netherlands. All responses were grouped in one of the three categories: low (practical and pre-vocational education), medium (higher general secondary education) to high education (pre-university education), using the standard classification of education in The Netherlands [34].

*Adolescents’ frequency of purchasing food and drinks in the school food environment and bringing these products from home.* To assess the frequency at which adolescents purchased food and drinks in and around their school, adolescents completed a short Food Frequency Questionnaire (FFQ). This FFQ was specifically developed for the aim of the present study. In this FFQ, adolescents were asked to report how frequently they purchased items within (1) FVS (e.g., fresh fruit and vegetables), (2) SSB (e.g., soft drinks and fruit juice), (3) SWS (e.g., cookies, candies, and ice cream), and (4) SAS (e.g., sausage roll, French fries, and pizza slice) at an average school week. This was asked on a 6-point scale, ranging from “never to almost never”(0 days per week) to “all schooldays” (5 days per week). For each of these categories, adolescents were asked to separately report the frequency at which they purchased items within this category from (1) their school canteen, (2) vending machines in school, or (3) at food retailers outside the school. The same FFQ was administered to assess the frequency at which adolescents brought food and drinks within these categories from home. 

*Demographic information mother.* Mothers’ age was obtained at baseline from the reported date of birth and the date of measurement, together with their educational level. Mothers also reported on their height in centimeters and weight in kilograms. Mothers’ BMI was calculated by dividing their weight in kilograms by their squared height in meters. Mothers’ educational level was measured with one item (‘what is your highest level of education?’), and response categories ranged from the lowest to the highest educational level in The Netherlands. All responses were grouped in one of the three categories: low (primary, lower secondary, lower vocational education), medium (higher secondary, vocational education) and high education (university of applied sciences, university), using the standard classification of education in The Netherlands [34].

*Adolescents’ perceived maternal relationship support.* The extent to which adolescents received relationship support from their mother was assessed by administering the short 12-item version of the Relationship Support Inventory (RSI; [35]). Adolescents indicated the degree to which they received emotional and instrumental support from their mother (e.g., “My mother supports what I do”) on a 5-point scale, ranging from 0 (absolutely not true) to 4 (absolutely true). The scores on the 12 items were summed and averaged to create a total score. Cronbach’s α in the present study was 0.85, indicating high internal consistency.

*Maternal monitoring.* Monitoring was assessed by asking mothers about their knowledge of their offspring’s daily activities [36]. Mothers indicated to what degree they were up to date on their offspring’s whereabouts (e.g., “Does your child need approval to leave the house at night”)? The three items existed of a 5-point scale, ranging from 0 (“never”) to 4 (“always”). The scores were summed and averaged to create a total score. Cronbach’s α in the present study was 0.77, indicating sufficient internal consistency.

### 2.4. Statistical Analyses

**Figure 1:**
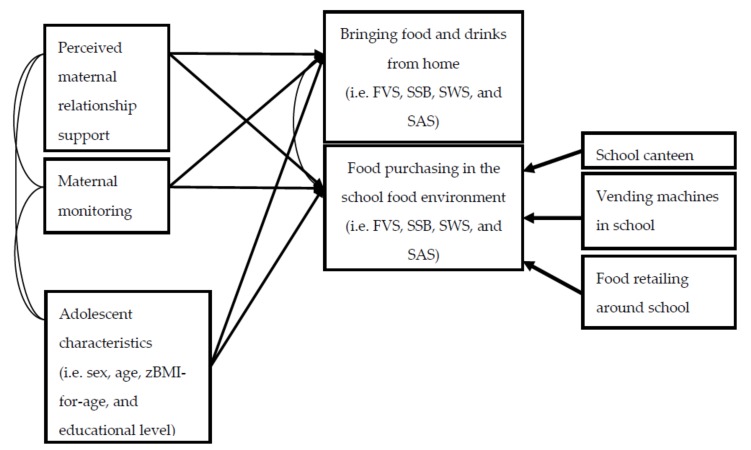
Conceptual model testing the associations between perceived relationship support, maternal monitoring and the frequency of bringing or purchasing food and drinks in the school food environment. Note. FVS = fresh fruit, vegetables and salad; SSB = sugar sweetened beverages; SWS = sweet snacks; SAS = savory snacks.

First, descriptive analyses were conducted to provide information about the sample and the frequency at which adolescents purchased food and drinks in their school food environment or brought these products from home. The second set of analyses existed of statistical tests to assess whether demographic factors relevant to the adolescent (i.e., sex, age, zBMI-for-age, and educational level) were associated with the frequency at which adolescents purchased and brought food and drinks. We conducted *t*-tests to assess sex differences, and ANOVAs with Games-Howell post-hoc comparisons were conducted to test differences between educational levels. Moreover, bivariate correlations for age and zBMI-for-age were conducted to assess whether these factors predicted any differences in the frequency to which adolescents purchased or brought food and drinks. These analyses were performed with SPSS version 24.0 (IBM Corp., Armonk, NY, USA). Finally, Structural Equation Modelling (SEM) in Mplus 8.0 was conducted [37] to assess whether maternal monitoring and relationship support were associated with the frequency of purchasing or bringing food and drinks. The latter existed of a latent variable constructed from the three contexts in which adolescents could purchase their food and drinks (i.e., school canteen, vending machines in school, or at food retailers outside the school). The model was estimated for all food categories simultaneously, i.e., FVS, SSB, SWS, and SAS. In a second model, we controlled for adolescents’ age, sex, zBMI-for-age and educational level. The model fit was assessed using the comparative fit index (CFI) and the Root Mean Square Error of Approximation (RMSEA). The CFI relates to the total variance accounted for by the model, where values close to 1, i.e., higher than 0.95, are considered adequate [38]. The RMSEA is based on the non-centrality parameter, where fit values of <0.06 are considered adequate. Full information maximum likelihood (FIML) procedures were used to account for missing data (e.g., in zBMI-for-age). We also report the Standardized Root Mean Square Residual (SRMR). Alpha was set at *p* < 0.05. See Figure 1 for the conceptual model of these analyses. The data that support the findings of this study are available from the corresponding author, upon reasonable request.

## 3. Results

### 3.1. Descriptive Analyses

Table 2 describes the frequency (days per week) to which adolescents (12–15 years) brought food and drinks within one of the four categories from home and how often they purchased items in these categories in their school food environment.

Overall, adolescents most frequently brought their food and drinks from home. Items within FVS, SSB and SWS were brought from home each about two days per week, on average. The frequency at which items within the categories were purchased in and around school was relatively low (each less than one day per week, on average).

### 3.2. Demographic Factors

We tested whether demographic factors (adolescents’ sex, age, zBMI-for-age and educational level) predicted any differences in frequency of bringing and buying food and drinks. The results of these analyses are displayed in Table 3 and Table 4. 

It was found that girls more frequently brought items within FVS from home compared to boys. Boys more frequently brought items within SSB from home and purchased these items more often from food retailers around school. Moreover, it was found that girls more frequently purchased items within SWS in their school canteen, whereas boys purchased items within SAS more often from food retailers around the school. Regarding educational level, it was found that boys and girls from lower educational levels reported a higher frequency of bringing and purchasing food and drinks. Specifically, they reported to purchase items within FVS more often from vending machines and food retailers around school. They also reported to bring and purchase items within SSBs and SAS more often than adolescents from medium or high educational levels. For more specific details, see Supplementary Tables S1 and S2.

Age was found to be positively associated with bringing and purchasing different types of food, indicating that older adolescents reported to bring and buy food and drinks more often than younger adolescents. zBMI-for-age was positively associated with purchasing items within FVS and SSB at food retailers around school, indicating that those with a higher zBMI-for-age purchased these food products more often around school. Adolescents with a lower zBMI-for-age reported to bring items within SWS less frequently from home

The associations between perceived maternal relationship support, maternal monitoring and frequency of bringing and purchasing food and drinks were assessed in Structural Equation Models. The total model showed an adequate fit (χ^2^_(df=78)_ = 115.03; CFI = 0.982; TLI = 0.966; RMSEA = 0.025; SRMR = 0.026).

### 3.3. The Role of Perceived Relationship Support

Relationship support was positively associated with bringing items within FVS from home, and negatively associated with purchasing items within SWS (see Table 5), indicating that more support from mothers was associated with bringing FVS more frequently and purchasing SWS less frequently.

In a next model, we added adolescents’ sex, age, zBMI-for-age, and educational level as covariates, again showing an adequate fit (χ^2^_(df=118)_ = 204.97; CFI = 0.966; TLI = 0.937; RMSEA = 0.032; SRMR = 031.). Although the association between perceived relationship support and purchasing SWS remained significant after adding these covariates, B = −0.129, SE = 0.059, *p* = 0.028, it became non-significant for bringing FVS from home, B = 0.059, SE = 0.038, *p* = 0.119, see Table 5.

### 3.4. The Role of Maternal Monitoring

Monitoring neither predicted frequency of bringing any food from home nor purchasing any food in or around school. Moreover, when adding covariates to the model, no significant associations were found (see Table 5).

## 4. Discussion

The present study aimed to get more insight into the frequency at which adolescents bring and purchase food and drinks in the school food environment. Specifically, we explored the potential associations between adolescents’ demographic information (i.e., sex, age, educational level, and zBMI-for-age) and their food purchasing behaviors. Furthermore, we investigated the associations between two specific parental factors (i.e., perceived relationship support from the mother and maternal monitoring) and adolescents’ food purchasing in and around their school.

One of the main findings of the present study is the observation that adolescents infrequently purchase food and drinks in and around their school. This is in line with previous work showing that self-purchasing of food and drinks in the school canteen or at vending machines in school is not very prevalent among Dutch adolescents [21,22]. Adolescents, however, also reported to infrequently visit food vendors near school. This finding is in contrast to previous studies which have suggested that adolescents more frequently spend money on food and drinks at food retailers around their school [21,22]. It should be noted, however, that the adolescents participating in this study were younger than those in previous work. An increase in food purchases in the school food environment with age may be due to an increased level of personal autonomy, greater access to own money and greater freedom to make choices about what to purchase and consume [39,40]. Indeed, Dutch adolescents aged 12-14 years receive 15-20 euros (which equals an amount of 16.5–22 US dollars) per month from their parents, and their budget increases with age as they may also generate income by means of a holiday or secondary job [41]. In future research, it would be worthwhile to further explore how adolescents’ food purchasing behavior in the school food environment may increase with age, linking current data with future waves of the longitudinal lifestyle cohort study [31,32]. This also holds for potential differences between boys and girls, and those with a lower versus higher educational background, as these were also found to be associated with adolescents’ food purchases in the school food environment.

Secondly, it was found that adolescents brought food and drinks mostly from home. This suggests that the availability of specific food and drinks in the home context plays a role in adolescents’ consumption behavior for the simple reason that these products are (freely) available to them. This is consistent with recent reviews that have summarized the importance of the availability and accessibility of healthy (i.e., fruit and vegetables) and unhealthy foods (i.e., SSB and energy-dense snack food) in the home context as a consistent predictor of desirable and undesirable food consumption in children and adolescents [25,42].

Thirdly, we found that the degree of relationship support adolescents perceived from their mother predicted the frequency of bringing and purchasing food and drinks. Specifically, it was found that those who perceived more support from their mother indicated to bring more FVS from home to school, and to purchase SWS less frequently in and around school. Although this is the first study to investigate this specific relationship, results are in line with suggestions that the quality of the parent-adolescent relationship can have an impact on the development of adolescent health risk behaviors [43]. Such research, for instance, has demonstrated that parental warmth, involvement, and emotional support are positively associated with higher fruit and vegetable consumption and lower intakes of high fat and/or sugar food and beverage intake [44,45]. We propose that higher levels of perceived relationship support may be the result of an authorative parenting style, and in this way play a protective role in adolescents’ food purchasing patterns in the school food environment. However, this is a posthoc suggestion and is speculative, so it will need to be empirically tested. Furthermore, although directions were in accordance with theory [27,29], the strengths of these associations were small. This may be explained by the notion that perceived support, as an indicator of general parenting style may be modeled at a more distal level of influence rather than at a more direct level of influence [46]. Finally, it should be noted that the relationship between perceived support and bringing FVS became non-significant when we controlled for demographic factors. This once again underscores the importance of these factors in this research area and further work is needed to gain more insight in their working mechanism.

Finally, this study found no evidence for a relationship between maternal monitoring and adolescents’ food purchasing patterns. Most research in this area has used the Child Feeding Questionnaire [47] or specific parenting style dimensions such as parents’ perceived strictness as measures of parental monitoring [48,49]. In this study, however, another monitoring scale was used [35]. This scale did not include specific items related to their child’s dietary behavior or food purchasing patterns in and around school, and therefore might have been too general. Likewise, this general monitoring scale might not have been sensitive enough to detect any differences between mothers. As this study is the first attempt to examine the association between maternal monitoring and adolescents’ food purchasing behavior in and around school, our results should be replicated to assure that our measures and results are valid and reliable.

Strengths of the presents study include its relatively large sample size and the inclusion from multiple informants (mothers and adolescents) which enabled us to assess the variables of interest from the most relevant source. Also, we examined adolescents’ frequency of food purchases in and around school, which is conceptually more directly linked to food environment exposure than dietary intake [50]. However, the present work also had limitations. First, data were cross-sectional, which limits us from drawing conclusions about the direction and temporality of the associations found. Future research is needed to examine the causal and longitudinal influence that perceived relationship support and monitoring might have on adolescents’ food purchasing behaviors. Second, our measures were based on self-reports of bringing and purchasing food and drinks in and around school within four different food and drink categories. This method may be biased due to social desirability and lack of specificity [51]. As a result, this study does not give insight into which specific food and drink items are most frequently brought or purchased by adolescents in and around school. Novel smart technologies make it possible to overcome some of these limitations by incorporating functions such as global positioning (GPS) and ecological momentary assessment (EMA) (cf. [50]). Future research may benefit from using these methodologies to examine adolescents’ actual food purchases, considering the accessible food environment in and around school by using a Geographical Information System (GIS) and on-site observations. This also makes it possible to examine socioeconomic differences in food outlet availability and its impact on adolescents’ food purchasing behaviors. A final limitation is that our study relied on a healthy sample of well-educated adolescents and their mothers with a homogenous cultural background. Therefore, our findings may not generalize to adolescents with a more at-risk background, such as those with an ethnic background or those whose parents have a lower socioeconomic position. Further research is required to establish potential differences between these adolescent subgroups in their food purchasing behaviors.

In The Netherlands, there is no compulsory system of school meals. Instead, adolescents may choose to bring their own food and drinks and/or to purchase (supplementary) items in the school food environment. Although self-purchasing of food and drinks in and around school was not very prevalent in this age group, the associations found with sex, age and educational level indicate that the school food environment remains an important area for preventive programs aimed at stimulating healthy dietary intakes among adolescents. Indeed, a recent review and meta-analysis demonstrated the positive effects of specific school food environment policy interventions on adolescent’s dietary intake behaviors [52]. Social norms regarding healthy eating may be a powerful mechanism underlying this effect [53,54]. It has been shown, for instance, that adolescents’ snack and soft drink consumption are highly associated with that of their peers [55], particularly when these food and drinks are highly available and accessible in their school environment [56]. By increasing the accessibility and availability of healthy food (and subsequently decreasing the supply of unhealthy food), adolescents are nudged towards making healthy food choices in and around school [14,15,16,17]. As a result of these changes, social norms around eating in and around school may change, thereby further increasing the effectiveness of these preventive programs. The findings of this study, however, also indicate that parents have an important role in providing their children with healthy food during school hours. This suggests that family-home determinants of healthy and unhealthy eating are important factors to consider when examining the impact of the school food environment on adolescents’ food purchasing patterns (cf. [57,58]).

## Figures and Tables

**Table 1:** Sample characteristics.

		Adolescents	Mothers
Age; Mean (*SD*)		13.78 (0.49)	45.05 (4.45)
Sex	Boys	46%	-
	Girls	54%	
Educational level	Low	39.1%	11.1%
	Medium	27.4%	46.3%
	High	33.5%	42.6%
BMI; Mean (*SD*)		zBMI-for -age -0.33 (1.02)	24.62 (4.17)
Relationship support (0–4)		3.32 (0.45)	-
Monitoring (0–4)		3.83 (0.44)	-

Note. zBMI-for-age is the measure that can be used from age 2 to 20 years to screen for obesity, overweight, or underweight. Body Mass Index (BMI).

**Table 2:** Means and standard deviations (between brackets) of bringing and purchasing food and drinks in the school food environment, in days per school week (*n* = 716).

	FVS	SSB	SWS	SAS
Bringing from home	2.04 (2.08)	1.83 (2.11)	2.59 (2.06)	0.28 (0.77)
Purchasing at school canteen	0.17 (0.73)	0.28 (0.86)	0.46 (0.97)	0.36 (0.80)
Purchasing at vending machines in school	0.07 (0.50)	0.24 (0.73)	0.46 (0.83)	0.15 (0.53)
Purchasing at food retailing around school	0.10 (0.51)	0.33 (0.81)	0.51 (0.89)	0.38 (0.76)

Note. FVS = fresh fruit, vegetables and salad; SSB = sugar sweetened beverages; SWS = sweet snacks; SAS = savory snacks.

**Table 3:** Differences in sex and educational level in the extent to which adolescents bring and purchase food and drinks the school food environment.

	Bringing from Home		School Canteen		Vending Machine in School		Food Retailing around School	
**Sex ^a^**		***p***		***p***		***p***		***p***
FVS	1 < 2	<0.001	NS	0.139	NS	0.934	NS	0.519
SSB	1 > 2	<0.001	NS	0.104	NS	0.586	1 > 2	0.009
SWS	NS	0.346	1 < 2	0.036	NS	0.099	NS	0.501
SAS	NS	0.572	NS	0.711	NS	0.414	1 > 2	0.024
**Educational level ^b^**								
FVS	NS	0.843	NS	0.177	1>2	0.018	1 > 3	0.013
SSB	1,2 > 3	<0.001	1 > 2,3	<0.001	1>2,3	<0.001	1 > 2,3	<0.001
SWS	NS	0.177	1 > 2,3	<0.001	1>3	0.012	1 > 2,3	<0.001
SAS	1,2 > 3	<0.001	1,2 > 3	0.001	1>3	0.002	1 > 3	<0.001

Note. ^a^ Significant differences are reported by indicating differences between boys (**1**) and girls (**2**). ^b^ Significant differences are reported by indicating differences between low (**1**), medium (**2**) and high (**3**) levels of education. Alpha is set at *p* < 0.05. NS is non-significant.

**Table 4:** Bivariate associations between adolescents’ age and zBMI-for-age with bringing and purchasing food and drinks in the school food environment.

	Bringing from Home		Purchasing in School Canteen		Purchasing at Vending Machine in School		Purchasing at Food Retailing around School	
**Age**	***r***	***p***	***r***	***p***	***r***	***p***	***r***	***p***
FVS	−0.05	0.215	0.05	0.153	0.09	0.023	0.04	0.239
SSB	0.11	0.002	0.08	0.034	0.02	0.653	0.09	0.014
SWS	0.05	0.156	0.11	0.004	0.05	0.151	0.08	0.036
SAS	0.14	<0.001	0.09	0.017	0.04	0.289	0.09	0.012
**zBMI-for-age**	***r***	***p***	***r***	***p***	***r***	***p***	***r***	***p***
FVS	0.04	0.382	0.03	0.435	0.06	0.160	0.10	0.026
SSB	−0.02	0.725	0.01	0.850	0.09	0.029	0.09	0.028
SWS	−0.09	0.032	−0.01	0.816	0.01	0.789	0.04	0.327
SAS	−0.03	0.559	−0.03	0.557	0.06	0.197	0.05	0.240

Note: Alpha is set at <0.05.

**Table 5:** Between perceived relationship support, maternal monitoring and the frequency of bringing and purchasing food and drinks.

	**Total Model**				**Total Model with Covariates**			
	**Bringing**		**Purchasing ^a^**		**Bringing**		**Purchasing ^a^**	
**FVS**	*B(SE)*	*p*	*B(SE)*	*p*	*B(SE)*	*p*	*B(SE)*	*p*
Relationship Support	0.081 (0.037)	0.028	−0.018 (0.044)	0.686	0.059 (0.038)	0.119	−0.009 (0.050)	0.861
Monitoring	0.003 (0.038)	0.941	0.020 (0.036)	0.568	−0.004 (0.036)	0.914	0.017 (0.034)	0.623
Sex	--		--		0.168 (0.037)	<0.001	0.033 (0.048)	0.489
Age	--		--		−0.027 (0.038)	0.480	0.074 (0.045)	0.102
Educational level	--		--		0.014 (0.037)	0.705	−0.117 (0.035)	0.001
zBMI-for-age	--		--		0.035 (0.040)	0.388	0.084 (0.063)	0.186
**SSB**								
Relationship Support	−0.032 (0.036)	0.374	−0.087 (0.057)	0.131	0.004 (0.035)	0.901	−0.062 (0.058)	0.288
Monitoring	−0.060 (0.043)	0.159	−0.010 (0.045)	0.823	−0.050 (0.039)	0.200	−0.007 (0.042)	0.861
Sex	--		--		−0.181 (0.037)	<0.001	−0.078 (0.045)	0.085
Age	--		--		0.077 (0.038)	0.042	0.032 (0.047)	0.497
Educational level	--		--		−0.130 (0.036)	<0.001	−0.232 (0.036)	<0.001
zBMI-for-age	--		--		−0.017 (0.042)	0.691	0.090 (0.063)	0.151
**SWS**								
Relationship Support	0.003 (0.038)	0.929	−0.135 (0.058)	0.021	0.005 (0.039)	0.903	−0.129 (0.059)	0.028
Monitoring	−0.003 (0.037)	0.953	−0.038 (0.045)	0.462	0.001 (0.035)	0.979	−0.034 (0.046)	0.464
Sex	--		--		0.040 (0.038)	0.292	0.109 (0.049)	0.027
Age	--		--		0.054 (0.038)	0.158	0.067 (0.045)	0.130
Educational level	--		--		−0.009 (0.038)	0.806	−0.234 (0.039)	<0.001
zBMI-for-age	--		--		−0.093 (0.043)	0.033	−0.010 (0.058)	0.858
**SAS**								
Relationship Support	−0.078 (0.043)	0.072	−0.061 (0.045)	0.168	−0.058 (0.043)	0.183	−0.045 (0.047)	0.343
Monitoring	0.021 (0.029)	0.473	−0.048 (0.049)	0.324	0.024 (0.028)	0.384	−0.045 (0.046)	0.329
Sex	--		--		−0.008 (0.037)	0.829	−0.004 (0.047)	0.934
Age	--		--		0.101 (0.044)	0.021	0.061 (0.049)	0.212
Educational level	--		--		−0.151 (0.030)	<0.001	−0.198 (0.039)	<0.001
zBMI-for-age	--		--		−0.031 (0.036)	0.391	0.055 (0.058)	0.342

Note. Effects are standardized Beta’s (standard errors in brackets); Except for monitoring, all variables were reported by the adolescents; ^a^ latent variable constructed from three items (i.e., purchasing in the school canteen, vending machine, or at food retailing around school. Alpha is set at <0.05.

## Supplementary Materials

The following are available online at https://www.mdpi.com/2072-6643/12/3/733/s1, Table S1: Means and Standard Deviations by Sex in the Frequency of Bringing Food From Home and Purchasing Food (i.e., in the School Canteen, Vending Machines and Around School). Table S2: Means and Standard Deviations by Educational Level, Testing Differences in the Frequency of Bringing Food From Home and Purchasing Food (i.e., in the School Canteen, Vending Machines and Around School). The data and questionnaire used in this study are available from the corresponding author upon request.
