# Supplementary material for: Adolescents’ Food Purchasing Patterns in The School Food Environment: Examining the Role of Perceived Relationship Support and Maternal Monitoring
Source: Nutrients. 2020 Mar 11;12(3):733. doi: 10.3390/nu12030733 (PMC7146524; doi:10.3390/nu12030733)
Supplement: Supplementary file 1 [file nutrients-12-00733-s001.pdf]

Table S2a

Means and Standard Deviations by Sex in the Frequency of Bringing Food From Home and Purchasing Food (i.e., in the School Canteen, Vending Machines and Around School).

| | Boys (1) | Girls (2) | t-value | *p* | Order |
|---|---|---|---|---|---|
| Bringing | | | | | |
| FVS | 1.63 (2.01) | 2.37 (2.09) | -4.81*** | <.001 | 1<2 |
| SSB | 2.27 (2.21) | 1.48 (1.96) | 5.04*** | <.001 | 1>2 |
| SWS | 2.51 (2.09) | 2.66 (2.02) | -.94 | .346 | NS |
| SAS | .30 (.81) | .26 (.73) | .61 | .572 | NS |
| Canteen | | | | | |
| FVS | .13 (.62) | .25 (.80) | -1.44 | .139 | NS |
| SSB | .34 (.99) | .23 (.75) | 1.67 | .104 | NS |
| SWS | .38 (.91) | .53 (1.02) | -2.11* | .036 | 1<2 |
| SAS | .37 (.75) | .35 (.85) | 1.19 | .711 | NS |
| Vending machine | | | | | |
| FVS | .07 (.53) | .07 (.53) | -.08 | .934 | NS |
| SSB | .25 (.71) | .22 (.75) | .54 | .586 | NS |
| SWS | .40 (.79) | .50 (.86) | -1.65 | .099 | NS |
| SAS | .13 (.45) | .17 (.58) | -.204 | .414 | NS |
| Around school | | | | | |
| FVS | .09 (.45) | .11 (.56) | -.64 | .519 | NS |
| SSB | .42 (.89) | .26 (.73) | 2.65** | .009 | 1>2 |
| SWS | .54 (.91) | .49 (.88) | .67 | .501 | NS |
| SAS | .45 (.77) | .32 (.44) | 2.83* | .024 | 1>2 |

Note: Significant differences reported by indicating differences between boys (1) and girls (2); *p<.05; **p<.01; ***p<.001.

Table S2b

Means and Standard Deviations by Educational Level, Testing Differences in the Frequency of Bringing Food From Home and Purchasing Food (i.e., in the School Canteen, Vending Machines and Around School).

| | Low (1) (n=279) | Medium (2) (n=196) | High(3) (n=241) | F-value | Order (*p*)[a] |
|---|---|---|---|---|---|
| Bringing | | | | | |
| FVS | 1.98 (1.99) | 2.05 (2.13) | 2.09 (2.15) | .17 | NS |
| SSB | 2.10 (2.09) | 2.03 (2.20) | 1.37 (2.00) | 9.09*** | 1>3 (p<.001); 2>3 (p=.004) |
| SWS | 2.54 (2.02) | 2.82 (2.03) | 2.46 (2.06) | 1.73 | NS |
| SAS | .42 (.95) | .28 (.77) | .11 (.42) | 10.93*** | 1>3 (p<.001); 2>3 (p=-016) |
| Canteen | | | | | |
| FVS | .23 (.79) | .16 (.74) | .12 (.63) | 1.74 | NS |
| SSB | .47 (1.11) | .21 (.70) | .11 (.57) | 12.30*** | 1>3 (p=.005); 1>3 (p<.001) |
| SWS | .68 (1.16) | .43 (.72) | .23 (.67) | 14.44*** | 1>2 (p=.014); 1>3 (p<.001) |
| SAS | .45 (.86) | .42 (.90) | .20 (.61) | 7.52** | 1>3 (p<.001); 2>3 (p=.007) |
| Vending machine | | | | | |
| FVS | .14 (.71) | .03 (.26) | .04 (.33) | 4.02* | 1>2 (p=.044) |
| SSB | .38 (.95) | .16 (.56) | .13 (.50) | 8.60*** | 1>2 (p=.011); 1>3 (p<.001) |
| SWS | .56 (1.01) | .43 (.72) | .35 (.65) | 4.45* | 1>3 (p=.011) |
| SAS | .23 (.71) | .13 (.40) | .07 (.31) | 6.21** | 1>3 (p=.002) |
| Around school | | | | | |
| FVS | .17 (.65) | .08 (.43) | .05 (.37) | 4.38** | 1>3 (p=.015) |
| SSB | .50 (1.01) | .26 (.64) | .19 (.62) | 10.54*** | 1>2 (p=.005); 1>3 (p<.001) |
| SWS | .69 (1.03) | .45 (.76) | .45 (.76) | 9.88*** | 1>3 (p=.009); 2>3 (p<.001) |
| SAS | .51 (.91) | .35 (.67) | .25 (.67) | 7.97*** | 1>3 (p<.001) |

Note: [a]Post-hoc comparisons using the Games-Howell procedure were used to identify significant pairs of group means; [b]p-values for post-hoc group mean differences were reported.
